# Chronic Glomerular Thrombotic Microangiopathy in a 72-Year-Old Patient with B-Cell Chronic Lymphocytic Leukemia and IgG Lambda Paraprotein

**DOI:** 10.3390/ijms262110310

**Published:** 2025-10-23

**Authors:** László Bitó, Timea Gurbity Pálfi, Krisztina Jost, Simon Péter Nagy, Zoltán Prohászka, Béla Iványi

**Affiliations:** 1 Department of Internal Medicine, Albert Szent-Györgyi Medical School, University of Szeged, 6722 Szeged, Hungary; bito.laszlo.zsolt@med.u-szeged.hu (L.B.);; 2 Institute of Laboratory Medicine, Albert Szent-Györgyi Medical School, University of Szeged, 6725 Szeged, Hungary; jost.krisztina@med.u-szeged.hu; 3 George Füst Complement Diagnostic Laboratory and Research Laboratory, Department of Internal Medicine and Hematology, Semmelweis University, 1088 Budapest, Hungary; nagy.simon.peter@semmelweis.hu (S.P.N.); prohaszka.zoltan@semmelweis.hu (Z.P.); 4 Department of Pathology, Albert Szent-Györgyi Medical School, University of Szeged, 6725 Szeged, Hungary

**Keywords:** chronic lymphocytic leukemia, CLL, classical pathway of complement, glomerular disease, monoclonal IgG lambda, monoclonal gammopathy of renal significance, kidney biopsy, nephrotic syndrome, thrombotic microangiopathy, TMA

## Abstract

The cause of nephrotic–nephritic syndrome and elevated blood pressure values was investigated by renal biopsy in a 72-year-old Caucasian male with B-cell chronic lymphocytic leukemia (B-CLL) and a low level of IgG/lambda paraprotein. Double-contoured glomerular capillaries, glomerular thrombi, interstitial B-CLL infiltrates, and normal-looking arteries and arterioles were observed histologically. The glomerular capillaries displayed nonspecific entrapment of IgM and C3 and pseudolinear C4d positivity immunohistochemically. With electron microscopy, diffusely effaced foot processes, widened and duplicated glomerular basement membrane (BM), mesangial cell interposition, and thickened, non-fenestrated, and serrated endothelial cells located on subendothelial BM layer(s) were seen. The peritubular capillaries lacked any significant BM multilayering. Chronic glomerular thrombotic microangiopathy (TMA) was diagnosed; the C4d positivity result indicated structural remodeling of glomerular capillary walls. Laboratory features of microangiopathic hemolytic anemia were absent. The functional complement assay found selective classical pathway activation and the consumption of early complement components. The components of the alternative pathway were not consumed. A disease-causing variant in the coding region of the complement *C2* gene was screened, with negative results. The kidney function gradually deteriorated to stage 4 chronic kidney disease over a period of six months. Second-line treatment with ibrutinib markedly decreased the leukemic symptoms, stopped the production of paraprotein, and eliminated the nephrotic syndrome; the kidney function improved. The decreased activity of the classical pathway remained unchanged. The culprit of glomerular anomalies seemed to be the paraprotein, which acted as a nephrotoxic mediator and triggered glomerular TMA. A hypothetical pathophysiologic explanation of TMA is presented. The paraneoplastic classical pathway activation of complement did not play any role in the development of glomerular TMA.

## 1. Introduction

Thrombotic microangiopathy (TMA) is the umbrella term for clinical syndromes with microvascular endothelial injury and thrombosis, tissue ischemia, and subsequent end-organ damage. The etiologic factors of endothelial injury are diverse [1]. TMA manifests itself in thrombocytopenia, microangiopathic hemolytic anemia (MAHA), proteinuria and/or hematuria, acute renal failure, and other abnormalities. The renal manifestation of acute TMA is characterized histologically by distinct glomerular and arterial/arteriolar changes and acute tubular epithelial cell damage. The duplication of glomerular basement membrane (GBM), focal segmental or global glomerulosclerosis, arterial intimal fibrosis, and interstitial fibrosis together suggest chronic TMA [2]. It may happen that the laboratory abnormalities of MAHA, acute kidney injury, and thrombocytopenia cannot be identified in patients with renal biopsy-proven TMA, and the diagnosis is classified as renal-limited TMA [1,3].

Monoclonal gammopathies may be associated with TMA [4,5,6]. Complement (C) studies identified low C3, normal C4, and high soluble C5b-9 levels in 33%, 100%, and 77% of tested patients, and this suggested a contribution of the alternative and terminal complement pathways to endothelial injury [6]. TMA is infrequently encountered in patients with B-cell chronic lymphoid leukemia (B-CLL). In the retrospective study by Strati et al. from the Mayo Clinic on CLL-associated renal diseases [7], 49 patients out of 4024 with CLL or monoclonal B-cell lymphocytosis underwent kidney biopsy for renal insufficiency or nephrotic syndrome, and TMA was diagnosed in six patients. All of them displayed acute kidney injury, proteinuria, hemolysis, low haptoglobin level, anemia, elevated lactate dehydrogenase (LDH), and schistocytes on a peripheral blood smear. The acute and systemic TMA appeared to be secondary to either CLL treatment or potential CLL complications. Two recent case reports, however, indicate that a direct pathogenetic link may exist between B-CLL and TMA, although the pathophysiologic mechanism of TMA remained unclear.

In the publication of Ma et al. [8], a 59-year-old woman with B-CLL, recurrent TMA, cryoglobulinemia, monoclonal serum IgM/kappa, MAHA, acute kidney injury, and massive proteinuria was evaluated via a renal biopsy. Three concurrent lesions were noted: mucoid intimal hyperplasia in interlobular arteries and glomerular endothelial swelling, indicative of acute TMA, mesangial proliferative C3 glomerulonephritis, and renal interstitial CLL infiltration. Glomerular capillary thrombi, cryoglobulin formation, and vasculitis were not seen. The administration of an inhibitor of Bruton’s tyrosine kinase, ibrutinib, induced a complete remission, the cessation of monoclonal IgM/kappa and cryoglobulin production; the serum C4 complement level and the lymphocyte count returned to the normal range. The case was concluded as an example of monoclonal gammopathy of renal significance (MGRS) associated with B-CLL [9]. The nephrotoxic monoclonal IgM/kappa paraprotein seemed to trigger complement C3 activation and induced TMA with C3 glomerulonephritis.

In the publication of Nasr et al. [10], a 72-year-old woman with B-CLL and type 2 diabetes displayed nephrotic syndrome and microhematuria. The kidney biopsy disclosed concurrent diabetic nephropathy and chronic TMA. Glomerular thrombi were not seen. The serum protein electrophoresis and serum immunofixation tests both proved to be negative. The complement functional panel testing revealed the dysregulated activity of C3 and C5 convertase and depleted classical and alternative pathways. The patient was negative for C3, C4, and C5 nephritic factors. Genetic testing for complement gene variants was similarly negative. As additional laboratory investigations revealed normal LDH, normal haptoglobin, normal bilirubin, no thrombopenia, and the absence of schistocytes, CLL-induced renal-limited TMA was concluded. Ibrutinib therapy led to a 90% reduction in the white blood cell count, a significant reduction in proteinuria, and complete resolution of edema. It was assumed that the B-CLL cells produced a nephrotoxic molecule that had remained unidentified using conventional laboratory methods, and it had somehow caused classical and alternative complement pathway dysregulation and subsequently renal-limited TMA.

Here, a 72-year-old male patient with B-CLL and IgG/lambda paraprotein is described who developed the nephrotic–nephritic syndrome in parallel with the worsening of leukemic symptoms. The renal biopsy evaluation revealed chronic glomerular TMA. Laboratory features of MAHA were sought, with negative results. Ibrutinib treatment halted the worsening of leukemic symptoms, eliminated the IgG/lambda paraprotein, and the nephrotic syndrome and glomerular microhematuria ceased. The report is the second in the English medical literature describing B-CLL, nephrotoxic IgG/lambda paraprotein, and renal-limited TMA. The novelty of the case is that the alternative pathway of the complement system was not involved in the development of TMA, and the TMA was exclusively confined to the glomerular capillary bed. The possible pathophysiology of TMA will be discussed.

## 2. Case Presentation

### 2.1. Medical History and Clinical Findings

A 72-year-old Caucasian male patient with B-cell chronic lymphocytic leukemia (B-CLL), nephrotic syndrome, dysmorphic hematuria, elevated blood pressure values, and decreasing renal function was admitted to our nephrology department for a renal biopsy evaluation.

His past medical history included mild and treated hypertension and hypothyroidism because of radioiodine ablation therapy for a toxic thyroid adenoma. In March 2023, a routine check of the laboratory values by his general practitioner revealed leukocytosis (13.45 G/L), lymphocytosis (7.31 G/L), smudge cells on a peripheral smear, normal hemoglobin content (142 g/L), mild thrombocytopenia (136 G/L), and normal serum protein and albumin levels. Regarding kidney function, there was a normal creatinine, blood urea nitrogen, and estimated glomerular filtration rate (eGFR) value; 2+ proteinuria and hematuria were found with a dipstick, and there were 14–15 erythrocytes in the urine sediment. The patient was referred to the hematology outpatient clinic.

In May, the hemogram demonstrated leukocytosis (13.85 G/L; absolute lymphocyte count: 9.11 G/L), mild anemia (hemoglobin 131 g/L), and thrombopenia (platelet 100 G/L), and there were smudge cells on the peripheral smear. No adenomegaly or hepatosplenomegaly was found during a physical examination. Fever, night sweats, and weight loss were absent. A flow cytometric investigation of peripheral blood found an abnormal lambda monotypic B-cell population suggestive of B-CLL ([cluster of differentiation: CD] CD19+, CD22 dim+, CD23 dim+, CD5+CD19+, CD5+CD23+, FMC7−; negative for myeloma cells [CD45−/CD19−/CD38+/CD56+/CD138+]). An LDH value of 288 U/L, hypoproteinemia (total protein 52 g/L), and hypoalbuminemia (31 g/L) were noted. The serum ions, creatinine, urea, eGFR, liver enzymes, and total serum bilirubin lay in the normal range. At this point, the “wait and see” strategy was adopted as the stage of B-CLL was classified as RAI 0 (Binet A).

In June, a pretibial edema developed bilaterally; the vascular surgeon could not find any vascular abnormality of the legs, and the cardiologist detected preserved myocardial function.

In August, the edema became generalized; there was a 15 kg gain in his body weight. Thoracic and abdominal CT scans demonstrated bilateral hydrothorax, massive ascites, mesenterial and retroperitoneal lymphadenomegaly, and mild splenomegaly (120 × 60 mm). Laboratory evaluations found anemia, thrombopenia, elevated LDH (400 U/L), serum total protein of 41 g/L, albumin of 23 g/L, cholesterol of 4.9 mmol/L, triglyceride of 1.5 mmol/L, eGFR of 49 mL/min/1.73m^2^, proteinuria of 6.7 g/day, and dysmorphic microhematuria. His blood pressure values were elevated, with the highest being 186/86 mm Hg.

**Figure 1:**
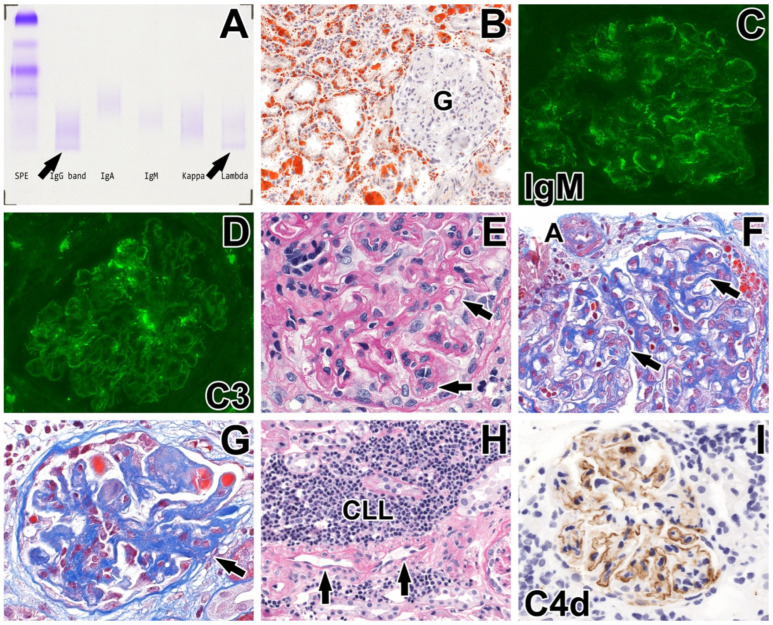
Summary of morphological findings. (**A**) The serum protein electrophoresis (SPE) demonstrated a monoclonal band in the gamma region. Immunofixation identified this protein as an IgG antibody paraprotein with a lambda light chain (arrows). The faint bands suggested a low concentration of paraprotein. (**B**–**I**) Renal biopsy findings. (**B**) A huge amount of reabsorbed lipid droplets in the tubular epithelial cells and interstitial cells. The glomerulus (G) is negative. Frozen section, Oil Red O, magnification X20. (**C**) IgM positivity with an imbibition pattern along the glomerular capillary loops (intensity: 1+). Direct immunofluorescence, X40. (**D**) C3 positivity with an imbibition pattern along the glomerular capillary loops and mesangium (intensity: 1+). Direct immunofluorescence, X40. (**E**) Double-contoured, thickened glomerular capillary loops (arrows) with cell interposition focally. Periodic acid–Schiff, X63. (**F**) Thickened glomerular capillary walls with double-contoured GBM (arrows). The afferent arteriole (A) appears normal. Crossmon’s trichrome, X63. (**G**) Glomerular thrombi (red), acellular closure of glomerular capillary loops with an attachment to the Bowman’s capsule (arrow). Crossmon’s trichrome, X40. (**H**) Focal CLL infiltrate in the periglomerular interstitial space. The interlobular artery (arrows) appears normal. Periodic acid–Schiff, X20 (**I**) Pseudolinear complement 4d positivity along the glomerular capillaries. Paraffin section immunohistochemistry, X40.

In September, he was admitted to the nephrology department for a detailed characterization of his hematologic disorder and renal biopsy investigation of the nephrotic–nephritic syndrome. The Yamshidi biopsy of bone marrow revealed CD20-positive diffuse infiltrates of B-CLL/small lymphocytic lymphoma in more than 80% of the bone marrow spaces (phenotype: CD20+, CD5+, CD23+, and CD200+; no abnormal p53 protein expression). The elements of normal hemopoiesis were conspicuously suppressed. An abnormal plasma cell population was not seen. A bone marrow FISH examination detected a gain of chromosome 1q21-22 in 62% of the specimens analyzed. A genetic test of peripheral blood identified a mutation of the *IgHV* gene, a gain of chromosome 12 (16%), no chromosome 17p deletion, and no *TP53* mutation. The serum electrophoresis revealed a faint abnormal band in the gamma region, and the immunofixation confirmed an IgG/lambda monoclonal component in the blood (see Figure 1A). The amount of free lambda light-chain molecules was more than twice the normal limit, and the free kappa/free lambda light-chain ratio was just at the lower border of the normal range. The serological work-up for autoantibodies (anti-nuclear antibody [ANA], anti-neutrophilic cytoplasmic antibody [ANCA], anti-phospholipase A2 receptor [anti-PLA2R] antibody, anti-thrombospondin type-1 domain-containing 7A [anti-THSD7A] antibody, anti-phospholipids), cryoglobulins, and viral panel (HBV, HCV, HIV, CMV, EBV) was negative. The complement levels C3 and C4 lay in the normal range. A renal ultrasound scan found normal-sized kidneys, and the corticomedullary border was indistinct. The laboratory abnormalities at the biopsy and 19 months later are summarized in Table 1.

### 2.2. Renal Biopsy Evaluation

**Figure 2:**
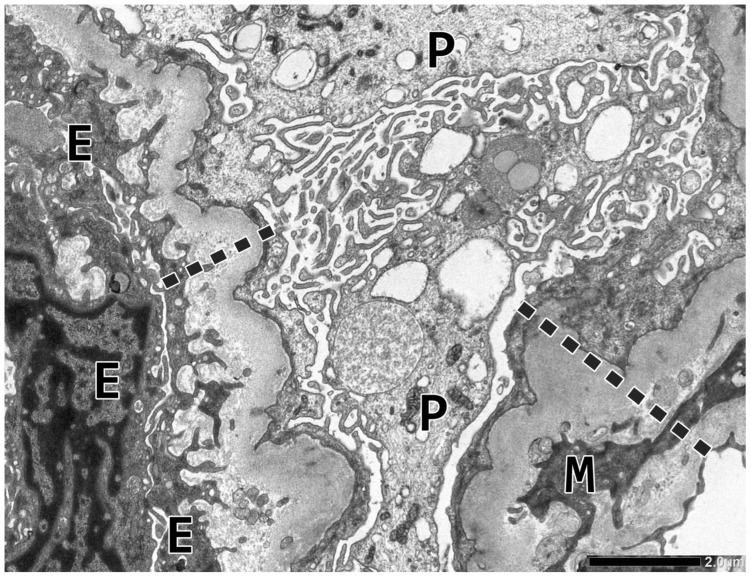
Electron microscopical abnormalities in glomerular capillaries. The podocyte (P) cell bodies underwent a microvillous transformation, and the foot processes are diffusely effaced. The capillary wall is irregularly and markedly thickened (dashed line), and the glomerular basement membrane is duplicated at the site of the mesangial cell (M) interposition. The widened lamina rara interna space displays invaginated endothelial cell (E) processes and new layers of subendothelial basement membrane. The endothelial cell layer is non-fenestrated. Magnification ×8000.

Since the clinical history and laboratory data suggested MGRS [9,11], a percutaneous renal biopsy was performed. The sample was investigated with direct immunofluorescence (FITC-conjugated IgG, IgA, IgM, C3, C1q, kappa, lambda, fibrinogen/fibrin; Dakopatts, Glostrup, Denmark) and Oil Red O staining on frozen sections, light microscopical special stains (hematoxylin and eosin, periodic acid–Schiff [PAS], Crossmon’s trichrome, acid fuchsin-Orange G, methenamine silver, and Congo red), and immunostainings for C4d (Cell Marque, Merck KGaA, Darmstadt, Germany) on formalin-fixed, paraffin-embedded tissue sections and electron microscopy. The main abnormalities observed are shown in Figure 1 and Figure 2.

Three normal-looking interlobular arteries, one globally sclerosed, and ten patent glomeruli (one with segmental sclerosis) were observed in the frozen sections. The glomerular capillary loops were thickened. The lumen of one glomerular capillary was clogged by hyaline thrombus (trichrome: blue). A significant amount of reabsorbed lipids was noted in the renal tubules and interstitial cells as a consequence of the nephrotic syndrome (Figure 1B). On immunofluorescence, the glomeruli were negative for IgG, IgA, kappa, and lambda light chains, C1q, and fibrinogen/fibrin. Diffuse-subglobal 1+ positivity of IgM (Figure 1C) and focal segmental 1+ positivity of C3 (pattern: imbibition; Figure 1D) were observed along the glomerular capillary loops.

Seven interlobular arteries, one globally sclerosed, and nineteen patent glomeruli (four with segmental sclerosis) were examined in the paraffin sections. One arterial profile exhibited mild intimal fibrosis, commonly seen in hypertensive aged patients. The arterioles did not show any abnormality (Figure 1F). The glomerular capillary loops were thickened, double-contoured (Figure 1E,F) and wrinkled, and thrombi occluded their lumen here and there. Some of the thrombi were red on trichrome staining (fibrin thrombi; Figure 1G). Focal segmental cell interposition contributed to the thickening of glomerular capillary walls. The mesangial regions were segmentally sclerosed, with the disappearance of capillary lumina and, on occasion, attachment to the Bowman’s capsule. Intracapillary leukocytosis (>5 leukocytes/glomerular profile), mesangiolysis, and fragmented red blood cells were not seen. C4d staining exhibited diffuse pseudolinear positivity of glomerular capillary walls (Figure 1I), and the intensity of staining varied from one glomerulus to another. For the arterioles and peritubular capillaries, C4d staining was negative. The interstitium was infiltrated by a few large foci of CD5- and CD19-positive and CD23-negative, cytologically atypical lymphoid cells (Figure 1H). Mild (15%) interstitial fibrosis and tubular atrophy completed the light microscopical alterations. The Congo red staining was negative.

Next, two glomeruli were examined electron microscopically. The podocyte foot processes were diffusely effaced. The glomerular capillary walls were markedly thickened due to the duplication of the glomerular basement membrane (GBM) and the presence of endothelial, mesangial, and podocytic cell processes in the widened lamina densa substance. The density of lamina densa was inhomogeneous and contained finely granular or membranous particles here and there. The endothelial cell layer was thickened, non-fenestrated, and serrated via cytoplasmic invaginations extending into the widened lamina rara interna space. The endothelial cell layer was frequently located on new BM layer(s) (Figure 2). The glomerular capillary lumina were narrowed or even clogged by platelets, swollen endothelial cells, and one or two mononuclear cells. The section plane was free of intracapillary thrombi.

Renal biopsy abnormalities at a glance are shown in Table 2.

All the peritubular capillaries (PTCs) present around the glomeruli were sampled for PTC basement membrane multilayering (PTCBML), as described in [12]. In brief, circumferential PTCBML was defined when the new BM layer was observed in more than 60% of the capillary circumference. The multilayering was read as the counts of PTCs with one or two layers, three or four layers, or five or six layers. Then the mean number of circumferential layers (PTC_CIRC_) was calculated by dividing the total number of circumferentially layered PC profiles (PTC_1−2_ × 2 + PTC_3−4_ × 4 + PTC_5−6_ × 6) by the total number of PTC profiles. Altogether 17 PTCs were analyzed. One profile exhibited three to four circumferential layers, five had one or two layers, and eleven appeared normal. The calculated PTC_CIRC_ value was 0.82, which did not indicate significant PTCBML.

### 2.3. Follow-Up and Therapy

The blood chemistry results did not reveal any systemic features of MAHA because the level of haptoglobin was slightly increased, the serum bilirubin level was normal, the LDH level was just above the upper limit of normal, and schistocytosis was not observed. The anemia and thrombopenia were interpreted as the manifestations of B-CLL. Our investigations of the functional complement profile are detailed in Section 2.4.

**Figure 3:**
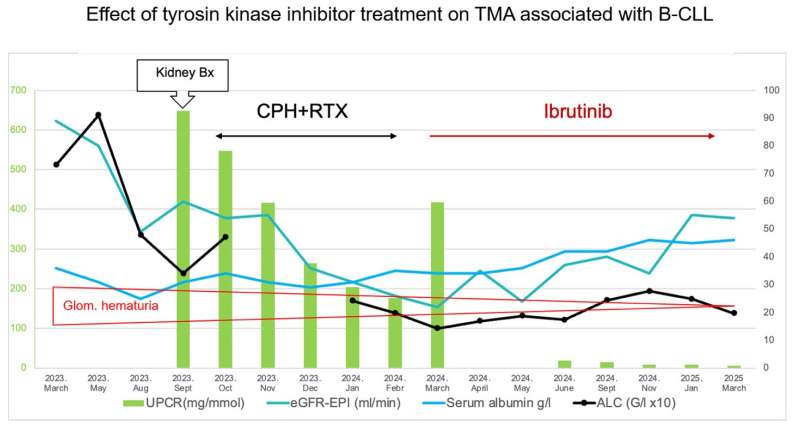
The ibrutinib therapy decreased leukemic lymphocytosis and eliminated the nephrotic syndrome, and the renal function improved. UPCR—Urine Protein Creatinine Ratio; eGFR—estimated glomerular filtration rate, ALC—absolute lymphocyte count, Bx—biopsy, CPH—cyclophosphamide, RTX—rituximab, Glom.—glomerular.

For the treatment of hematological disease, between October 2023 and February 2024, the patient received six cycles of rituximab 375 mg/m^2^ (CD20 monoclonal antibody) and four cycles of CVP (cyclophosphamide 750 mg/m^2^, vincristine 1.4 mg/m^2^- cap of 2 mg and 40 mg/m^2^ prednisolone taken orally once a day for 5 days). Since the lymphocytosis was reduced but the anemia, thrombocytopenia, and nephrotic syndrome persisted, the therapy was judged to be ineffective. For the second line treatment from April 2024, continuous ibrutinib 420 mg once a day was initiated. Due to progressive cytopenia, the dose was reduced to 280 mg once a day. The full blood count returned to normal six months later. An ultrasound scan of the abdomen revealed a normal-sized spleen and no adenomegaly. The kidney function improved (see Table 1 and Figure 3).

### 2.4. Complement Profile Assessment

#### 2.4.1. Methodology

Complement C1q (measured after June 2024), C3, C4, and haptoglobin levels were measured by nephelometry (BN II System, Siemens Healthineers, Erlangen, Germany). Total alternative and MBL-lectin pathway activities were assessed using commercially available kits (WIESLAB® Complement Alternative Pathway and Complement MBL Pathway, COMPLAP330 and COMPLMP320, Svar Life Science, Malmö, Sweden) based on the manufacturer’s instructions. Total classical pathway activity was tested using a hemolytic assay based on Mayer’s method, as previously described (PMID: 6492124; 37823685). Complement Factors I and B were measured by radial immunodiffusion (PMID: 21620101). The concentrations of C1q (measured before June 2024), Factor H, anti-Factor H, and anti-C1q antibodies were determined by in-house ELISA methods, as previously described (PMID: 21620101; 20181396; 10080837). The complement activation marker known as sC5b-9 was detected in EDTA plasma using a commercially available ELISA kit (MicroVue sC5b-9 Plus EIA, A029, QuidelOrtho, San Diego, CA, USA). ADAMTS-13 activity was determined via the FRET method (PMID: 21620101). *C2* gene screening was carried out by PCR amplification of the coding exons and flanking regions, followed by Sanger sequencing (BigDye™ Terminator v3.1 Cycle Sequencing Kit; 4337456, Applied Biosystems, Foster City, CA, USA). The primer sequences and PCR conditions are available upon request.

#### 2.4.2. Results (Table 3)

The first assay, performed three weeks after the renal biopsy evaluation, confirmed a normal ADAMTS13 function. The activity of the classical pathway and the levels of C1q and C4 were markedly lower. The level of C3 and the activation capacity of the alternative pathway were slightly lower. No antibodies against C1q or Factor H were detected. Levels of complement Factor H and Factor I were marginally elevated, while the Factor B level was within the normal range. The haptoglobin level was increased, which did not support intravascular hemolysis. Overall, the classic pathway activity and consumption of early complement components C1q and C4 were seen. The components of the alternative pathway were not consumed.

While the patient was on ibrutinib therapy, two follow-up assays were performed. Fifteen months after the renal biopsy investigation, the assay still demonstrated a severely decreased classic pathway activity. At 19 months, both the classic pathway and the MBL lectin pathways appeared to be deficient again, raising the possibility of C2 complement deficiency (the most frequent cause of classical pathway deficiency in Central Europe). Therefore, the genomic DNA of the patient underwent a polymerase chain reaction, and the complete coding region of the complement *C2* gene from exon 1 to exon 18 was investigated using Sanger sequencing. No potentially disease-causing variant was identified in the coding exons and their flanking regions. The results of the sequencing ruled out the most common inherited cause of decreased classical pathway activation.

**Table 3:** Complement abnormalities three weeks after the renal biopsy procedure and during the follow-up period (on ibrutinib). NT—not tested.

Complement Profile	3 Weeks After the Biopsy	15 Months Later	19 Months Later
Total complement activity, classical pathway (hemolytic test; CH50/mL; 48–103)	1	6	0
Total complement activity, alternative pathway (WIELISA-ALT; %; 70–125)	63	71	77
Total complement activity, MBL lectin pathway (WIELISA-MBL; %; 35–130%)	NT	NT	0
C1q antigen (mg/L; 60–180; from May 2024 mg/L; 150–320)	14	270	268
Complement C3 (g/L; 0.9–1.8)	0.79	1.45	1.54
Complement C4 (g/L; 0.15–0.55)	0.04	0.37	0.39
Complement Factor H antigen (mg/L; 250–880)	959	499	NT
Complement Factor I antigen (%; 70–130)	184	128	NT
Complement Factor B antigen (%; 70–130)	120	124	NT
Anti-H Factor IgG (Au/mL; <110)	5	3	NT
Anti-C1q IgG (mU/L; <52)	20	2	6
SC5B-9 (terminal complement complex (ng/mL; 110–252)	NT	426	NT
ADAMTS13 activity (%; 67–150)	70	79	NT
Haptoglobin (g/L; 0.3–2.0)	3.73	2.79	NT

### 2.5. Screening for Anti-Endothelial Cell Antibodies (AECAs)

The patient’s serum, taken at the time of the renal biopsy investigation and stored at −20 °C, was examined with a triple tissue indirect immunofluorescence (IIF) assay (Europlus LKS Mosaic, Biochip Technology, Euroimmun Medizinische Labordiagnostika AG, Lübeck, Germany) using a rat kidney, a rat stomach, and monkey liver tissues, as well as Hep-2 cells applied as substrates. FITC-labeled anti-human IgG (goat) was used as a conjugate. The Hep-2 mosaic test excluded the presence of anti-nuclear antibodies. In the rat kidney, the glomerular capillaries exhibited diffuse-global faint staining at a serum dilation of 1:10. This staining pattern was not observed 19 months later when the patient was on ibrutinib therapy. This doubtful positive IIF assay on the rat kidney was checked using frozen kidney sections obtained from an adult patient with renal cell carcinoma at a serum dilation of 1:10. The glomerular capillaries were unequivocally negative.

## 3. Discussion

The clinical work-up of kidney dysfunction strongly implied MGRS-associated kidney disease [9]. To our surprise, the renal biopsy findings excluded monoclonal immunoglobulin deposits and C3 glomerulopathy. The double-contoured glomerular capillaries, the electron microscopically observed non-fenestrated, thickened and serrated glomerular endothelial cells underlined by BM layer(s), and mesangial cell interposition led us to conclude the case as chronic TMA. The thrombi indicated activity of the condition. TMA can affect the small interlobular arteries and afferent arterioles, and it can cause the BM multilayering of peritubular capillaries [12]. The focused reading of these vessels for suggestive lesions of TMA, however, proved negative. The blood chemistry data did not reveal any features of MAHA. In summary, chronic TMA affecting just the glomerular capillaries was diagnosed in the background of nephrotic–nephritic syndrome. The second-line treatment with ibrutinib potently affected the leukemic lymphocytosis, regressed lymphadenomegaly and splenomegaly, and eliminated the paraprotein. Moreover, the nephrotic syndrome and microhematuria had ceased, and the kidney function parameters had improved (summarized in Figure 3). The positive and parallel response of hematologic and kidney disease to ibrutinib therapy strongly suggested a pathogenic link between the two disorders.

The morphologic diagnosis of glomerular TMA made it necessary to investigate whether the activation of the complement system contributed to the endothelial injury of glomeruli. A functional complement assay revealed an abnormally activated classic pathway, with the consumption of early complement components. The alternative pathway was not affected. These results contrasted sharply with those in the French study on monoclonal gammopathy-associated TMA, which reported an atypical hemolytic–uremic syndrome-like pathophysiology through alternative pathway and terminal complement pathway activation [6]. The renal biopsy findings in our patient excluded monoclonal immunoglobulin deposits, including cryoglobulin deposits, and in this way, antibody–antigen complexes binding to the C1q complex could not, therefore, be the cause of classical pathway activation. Chemotactic complement components were surely not generated in sufficient quantity because intracapillary leukocytosis was not the feature of glomerular TMA. The ultrastructural appearance of glomerular capillaries resembled that seen in chronic transplant glomerulopathy, and we wondered whether the paraprotein acted as an AECA. The IIF assay on human kidney tissue, however, was found to be negative. Hence, the possibility of abnormal classical pathway activation triggered by AECAs was ruled out. The positive GBM-C4d result was interpreted as an immunohistochemical sign of structural remodeling of the glomerular capillary walls [13,14]. Overall, immune complex deposition or AECA activity as activators of the classical pathway of complement were both excluded. Genetic testing of the complement *C2* gene did not identify a common inherited pathogenic variant as the cause of decreased classical pathway activation. It should be noted that even at the 19th month, control classical pathway deficiency was observed, raising the suspicion of complement deficiency (in other than the *C2* gene) or a functional interaction between the remaining paraproteins and complement.

The literature findings tell us that the classical pathway activation at a low level is a relatively frequent, albeit a probably non-harmful phenomenon in B-CLL, mediated by the formation of IgG hexamers and binding to alpha-2 macroglobulin [15,16,17]. Although we did not examine the possibility of IgG hexamers acting as an activator of the classical pathway, we do not think that the paraneoplastic classical pathway activation had anything to do with the development of glomerular TMA. Next, the theoretical pathophysiological events of glomerular TMA in our patient will be discussed.

The culprit of glomerular anomalies had to be the paraprotein molecule since the disappearance of IgG/lambda paraprotein was strongly associated with the cessation of nephrotic syndrome. The loss of fenestrae and thickening of endothelial cell bodies might be the footprint of cytokine mediation because a similar reaction pattern was noted in cytokine-stimulated peritubular capillary endothelial cells in cases with the acute T cell-mediated rejection of kidney allografts [18]. The resemblance of the two conditions with endothelial cell morphology gave us the idea that the paraprotein possessed cytokine features that interfered with the vascular endothelial growth factor (VEGF) signaling pathway between podocytes and glomerular endothelial cells. Generally, this pathway is critical in the maintenance of the glomerular filtration barrier.

Upon reviewing the literature on the role of VEGF signaling in the pathogenesis of renal-limited TMA, both stimulatory and inhibitory insults can result in renal TMA. Notably, the participation of the complement system in the development of glomerular changes is not required. In the very rare POEMS syndrome (polyneuropathy, organomegaly, endocrinopathy, monoclonal protein, skin changes), the M-protein is almost always lambda, and through molecular mimicry between monoclonal lambda light chains and some receptors involved in vascular endothelial growth factor (VEGF) secretion, specific lambda light chains induce VEGF hypersecretion and TMA-like glomerular changes [19,20,21]. In cancer patients treated with VEGF antibodies, such as bevacizumab, the prolonged and enhanced inhibition of the VEGF pathway most likely culminates in TMA-like glomerular microangiopathy. Many of the bevacizumab-treated patients exhibit massive proteinuria and hypertension, as well as renal insufficiency [22,23]. Although the clinical presentation of bevacizumab nephrotoxicity shared similarities with our case, the morphology of TMA in bevacizumab nephrotoxicity compared to our case was not the same. In conclusion, the precise pathophysiology of glomerular TMA in our patient remained hidden.

## 4. Conclusions

In this paper, a very rare example of MGRS-associated kidney disease was presented. TMA that only affected the glomeruli was diagnosed in a patient with B-CLL and IgG/lambda paraprotein. TMA was manifested clinically in an insidiously developing nephrotic–nephritic syndrome and elevated blood pressure values. Complement functional panel testing revealed an abnormal activation of the classical pathway, regarded as a non-harmful paraneoplastic event that had no link to the evolution of TMA. The testing, the laboratory data, and the clinical symptoms excluded alternate pathway activation-induced atypical hemolytic–uremic syndrome. Ibrutinib treatment improved the hematologic disorder, halted the paraprotein production, stopped the nephrotic syndrome, and improved the kidney function. It was concluded that the paraprotein acted as a nephrotoxic mediator, and it had triggered chronic TMA. The pathophysiology of the patient’s TMA remained clinically unresolved.

## Figures and Tables

**Table 1:** Laboratory abnormalities at the time of the renal biopsy procedure and during the follow-up period (on ibrutinib). FLC—free light chain, NT—not tested, SPEP—serum protein electrophoresis.

Blood Test	At Renal Biopsy	19 Months Later
Total protein (g/L; 60–87)	41	66
Albumin (g/L; 34–48)	23	46
Total bilirubin (μmol/L; <21.0)	5.1	8,9
Total cholesterol (mmol/L; <5.2)	4.9	5.28
Triglyceride (mmol/L; <1.70)	1.5	1.24
Creatinine (μmol/L; 71–115)	122	114
eGFR (ml/min/1.73 m^2^; >90)	50	54
White blood cell count (Giga/L; 5.00–10.00)	7.49	8.41
Lymphocyte count (Giga/L; 1.02–3.55)	4.34	1.97
Hemoglobin (g/L; 130–165)	97	133
Platelet (Giga/L; 150–400)	91	123
LDH (U/L)	238	NT
**Serological work-up for autoantibodies, cryoglobulins, and viral panel**	Negative	NT
**Monoclonal protein testing**		
SPEP, gamma-globulins (%; 6.2–15.4)	8.3; faint abnormal band in the gamma region	7.3; no extrafraction
Serum immunofixation	IgG/lambda monoclonal component	IgG/lambda suspected
Kappa FLC (mg/L; 6.70–22.40)	19.4	13.7
Lambda FLC (mg/L; 8.3–27.0)	61.1	23.2
Kappa/lambda ratio (0.31–1.56)	0.31	0.59
**Urine abnormalities**		
Albumin/creatinine ratio (mg/mmol; <2.50)	648	6.5
Dysmorphic red blood cells/visual field	40–50	6–8

**Table 2:** Summary of renal biopsy abnormalities. ATN—acute tubular necrosis, C3—complement 3, C4d—complement 4d, CLL—chronic lymphoid leukemia, DCs—double contoured glomerular capillaries, dGBM—doubled glomerular basement membrane, dFPE—diffuse foot process effacement.

No. of Glomeruli/ GS	Glom. Thrombi	MSG	DCs	IFTA (%)	Focal CLL Infiltr.	ATN	Arterial Thrombi/ Sclerosis	Arteriolar Thrombi	Lipids in Tubuli	IH	EM
24/1	Yes	No	Yes	15	Yes	No	No/mild intimal fibrosis in 1 profile out of 10	No	Yes	IgM, C3, C4d	dFPE, dGBM, MCI, thickened, serrated GECs

EM—electron microscopy of glomeruli, GECs—glomerular endothelial cells, Glom.—glomerular, GS—globally sclerosed, IFTA—interstitial fibrosis and tubular atrophy, IH—immunohistochemistry of glomeruli, infiltr.—infiltrates, MCI—mesangial cell interposition, MSG—mesangiolysis.

## Data Availability

Sequence datasets are unavailable due to privacy and ethical restrictions.
